# Isolation and identification of chlorate-reducing *Hafnia* sp. from milk

**DOI:** 10.1099/mic.0.001347

**Published:** 2023-07-14

**Authors:** William P. McCarthy, Meghana Srinivas, Martin Danaher, Christine O. Connor, Tom F. O. Callaghan, Douwe van Sinderen, John Kenny, John T. Tobin

**Affiliations:** ^1^​ Food Chemistry and Technology Department, Teagasc Food Research Centre, Moorepark, Fermoy, Cork, Ireland; ^2^​ School of Food Science and Environmental Health, Technological University Dublin, Grangegorman, Dublin 7, Ireland; ^3^​ Food Biosciences Department, Teagasc Food Research Centre, Moorepark, Fermoy, Cork, Ireland; ^4^​ School of Microbiology, University College Cork, Cork, Ireland; ^5^​ APC Microbiome Ireland, University College Cork, Cork, Ireland; ^6^​ Food Safety Department, Teagasc Food Research Centre, Ashtown, Dublin 15, Ireland; ^7^​ School of Food and Nutritional Sciences, University College Cork, Cork, Ireland; ^8^​ VistaMilk Science Foundation Ireland Research Centre, Teagasc, Moorepark, Fermoy, Cork, Ireland

**Keywords:** chlorate, contamination, dairy, food safety, *Hafnia*, milk

## Abstract

Chlorate has become a concern in the food and beverage sector, related to chlorine sanitizers in industrial food production and water treatment. It is of particular concern to regulatory bodies due to the negative health effects of chlorate exposure. This study investigated the fate of chlorate in raw milk and isolated bacterial strains of interest responsible for chlorate breakdown. Unpasteurized milk was demonstrated to have a chlorate-reducing capacity, breaking down enriched chlorate to undetectable levels in 11 days. Further enrichment and isolation using conditions specific to chlorate-reducing bacteria successfully isolated three distinct strains of *Hafnia paralvei *. Chlorate-reducing bacteria were observed to grow in a chlorate-enriched medium with lactate as an electron donor. All isolated strains were demonstrated to reduce chlorate in liquid medium; however, the exact mechanism of chlorate degradation was not definitively identified in this study.

## Introduction

Chlorate (ClO_3_^−^) is an emerging chemical residue of growing concern within the food and beverage industries [1, 2]. It is formed by the unavoidable decomposition of hypochlorite (ClO^−^) in concentrated solutions used for sanitation [3]. Chlorate can enter food through contact with chlorinated water (treated with hypochlorite) or residues from clean-in-place processes on food contact surfaces [4].

Chlorate is associated with several adverse health implications, including oxidation of the iron centre of haemoglobin to form methaemoglobin, and detrimental effects that it can have on growth and development due to inhibition of thyroid hormone synthesis [5–10]. It is noted that infants are a vulnerable group of particular concern due to infant haemoglobin being more easily oxidized than mature red blood cells, and the high levels of cellular growth and cognitive development occurring in this period for which the thyroid is essential [9, 11, 12]. By the age of 6 months, only 13 % of infants still rely entirely on breast milk [13, 14]. This high dependency of infants on dairy-based ingredients, and the adverse health implications of dietary chlorate, highlights the importance of improved management of chlorate levels in milk by the dairy industry [15]. Currently there is only one documented means of chlorate removal from milk that could be applied at an industrial scale [16], but there has been growing interest in the use of microbial enzymes as an alternative, biological mitigation strategy.

Microbial reduction of chlorine oxyanions under anaerobic conditions has been documented for over 70 years [17–23], though there is sparse information on the microbes that are capable of this metabolic conversion or on the biochemical pathways. Biologically mediated (per)chlorate reduction has been noted to occur in a wide range of environments and is associated with a class of micro-organisms called dissimilatory (per)chlorate-reducing bacteria (DPRBs), with many strains isolated and characterized in recent years contributing to a greater understanding of this phenomenon [17, 21, 24–28]. This reduction by DPRBs produces harmless chloride ions and has created much interest as a potential means of remediating (per)chlorate contamination in the environment [29]. Phylogeny analysis of known DPRBs indicates that they are exclusively found within the phylum *Proteobacteria* [26], possibly evolving from a common ancestor that was lost in multiple genera over time. Another theory suggests that the ability is a recent evolution and is present due to horizontal gene transfer events. The latter is supported by the lack of phytogenic synergy between the 16S rRNA and an essential gene in the (per)chlorate reduction pathway, chlorite dismutase (*cld*), in the organisms [24, 26, 30, 31].

**Fig. 1.**
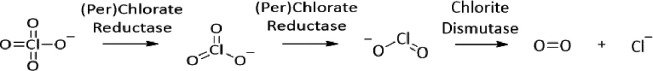
Reduction of perchlorate to chloride by using perchlorate reductase to reduce perchlorate and chlorate to chlorite, and further reduction to chlorine and oxygen.

The pathway that reduces perchlorate to chloride in DPRB involves the formation of chlorate and chlorite as intermediates, and is catalysed by the enzyme (per)chlorate reductase (*per*), responsible for the reduction of perchlorate and chlorate to chlorite, and further reduction to chloride and oxygen by the *cld* gene (Fig. 1) [32, 33].

Perchlorate and chlorate have been found in milk and other dairy products. Perchlorate is thought to enter the milk in the mammary gland due to the sodium iodide symporter during milk formation, allowing it to pass through the blood–milk barrier after consumption of contaminated water and feed [34–36], while chlorate is primarily associated with processing water and chlorinated detergent use [4, 37]. Studies have indicated that perchlorate in human breast milk has been found to be five times higher than the levels recorded in bovine milk [35], which is further supported by evidence that up to 80 % of perchlorate consumed by the cow is metabolized, indicating that the animal has developed some ability to metabolize the perchlorate it consumes [38]. It has been hypothesized that this reduction of perchlorate within the anaerobic environment of the cow’s rumen is due to microbes that carry out a series of enzymatic conversions as discussed previously, which has been further supported through the use of isotopically labelled chlorate in ruminal fluid [39].

DPRBs have been isolated from many environments, but efforts to isolate these micro-organisms from a matrix related to cows have been unsuccessful [39]. The current study investigated chlorate reduction in raw and heat-treated milk during low temperature storage, to determine whether this chlorate reduction was mediated by bacteria, and isolate and identify any chlorate-reducing bacteria present in raw milk.

## Methods

### Reagents and chemicals

Sodium chlorate solutions were acquired from Sigma and stored at 4 °C. Other chemicals used in the study were ammonium chloride (VWR), potassium phosphate dibasic anhydrous, sodium phosphate monobasic monohydrate, ferrous chloride tetrahydrate, sodium selenite, sodium molybdate dihydrate, nickel chloride hexahydrate, zinc chloride, manganese chloride tetrahydrate, cobalt (II) chloride hexahydrate, copper (II) chloride dihydrate, boric acid, hydrochloric acid, EDTA disodium salt dihydrate, sodium hydroxide, lactate, acetate, l-cysteine HCl (Sigma), Bacto agar, resazurin (Difco), milk plate count agar and maximum recovery diluent (Sigma).

### Initial screening for chlorate reduction in raw milk

Raw milk (3×10 l) was collected from three research farms (AGRIC, Moorepark, Fermoy, Cork, Ireland). The milk from each farm was evenly distributed among five 2 l sterile bottles. A chlorate solution was added to four samples to achieve a final concentration of 1.2 mM, with one aliquot kept as a chlorate-free control. The chlorate-free control and one enriched milk sample remained unpasteurized as non-treatment controls. One chlorate-enriched milk sample was incubated for 1 h at 37 °C, and two chlorate-enriched milk samples were heat treated using a Microthermics unit (UHT/HTST electric model 25 HV hybrid; Liquid Technologies). One was pasteurized at 73 °C for 15 s, and the other was ultra-high temperature (UHT) treated at 135 °C for 4 s as a sterile control. All treatments were performed within 3 h of milk collection. After treatment, milk samples were aseptically transferred into sterile tubes, stored at 4 °C, and sampled daily to allow chlorate and microbial analysis.

### Microbiological isolation procedure

#### Preparation of selective media

Bacteria with the ability to reduce chlorate were isolated using a nutrient solution designed by modification of the recipes of Prata *et al*. [40], Xu and Logan [41], and Kengen *et al*. [32]. A phosphate buffer was prepared to contain 1.55 g K_2_HPO_4_ l^−1^, 0.85 g NaH_2_PO_4_.H_2_O l^−1^, 0.25 g NH_4_Cl l^−1^, 0.1 g MgSO_4_.7H_2_Ol^−1^. A mineral solution was prepared by adding 10 ml HCl (37 %) l^−1^, 17 mg Na_2_SeO_3_ l^−1^, 1.5 g FeCl_2_.4H_2_O l^−1^, 0.36 g Na_2_MoO_4_.2H_2_O l^−1^, 2.4 g NiCl_2_.6H_2_O l^−1^, 0.1 g MnCl_2_.4H_2_O l^−1^, 0.19 g CoCl_2_.6H_2_O l^−1^, 2 mg CuCl_2_.2H_2_O l^−1^, 3 g H_3_BO_3_ l^−1^, 3 g EDTA l^−1^. All solutions were made using distilled, deionized (18.2 Ω) water. The selective media was prepared by adding 1 ml l^−1^ nutrient solution to the phosphate buffer solution and 0.5 mg resazurin l^−1^, with lactate or acetate as an electron donor and NaClO_3_ as an electron acceptor added in a molar ratio of 2 : 1.

The selective media (SM) was prepared anaerobically by boiling and sparging with N_2_ as 0.5 g l-cysteine HCl l^−1^ was added to reduce the resazurin from violet to pink. When the solution was cooled, the pH was adjusted to 7.2 with NaOH. A total of 10 ml of the solution was then transferred anaerobically into 18×150 mm hungate type anaerobic culture tubes (Bellco Glass) and immediately sealed with a 20 mm septum stopper with a tear-off aluminium seal crimped over it. The tubes were autoclaved at 121 °C for 15 min (ASB260BT; Astell). Selective media agar plates were made by adding 15 g Bacto agar l^−1^ to the selective media.

#### Isolation of chlorate respiring bacteria from milk

For isolation trials, one farm (Moorepark Fermoy, Cork) was selected for further investigation, isolation and identification of micro-organisms capable of chlorate degradation. Raw milk was collected from the bulk tank of the selected farm and subjected to one of four different enrichment conditions: (i) inoculated fresh into SM containing lactate and chlorate, (ii) inoculated fresh into SM containing acetate and chlorate. The remaining two enrichment conditions involved the initial addition of 100 mg chlorate l^−1^ to the milk and refrigeration for 10 days at 4 °C before inoculation into SM containing (iii) acetate and chlorate or (iv) lactate and chlorate. In all cases, 0.1 ml raw milk was added to the prepared 10 ml SM under anaerobic conditions in hungate tubes. Incubation was carried out at 37 °C under constant shaking.

A 2 mM ClO_3_^−^ concentration was selected to start the enrichment in SM. Over 1 month, continuous transfers (1 ml from older medium into 10 ml fresh sterile medium) were made in sterile conditions. During this period, the concentration of ClO_3_^−^ was increased from 2 to 6 mM. Cultures became turbid in roughly 7 days. After 14 days, the 6 mM chlorate inoculated tubes were plated on the SM agar containing lactate or acetate and incubated under anaerobic conditions at 37 °C for 7 days.

#### Colony selection and purification

Colonies were picked off the plates based on morphology and streak plated on their respective fresh agar, then purified by picking a colony from the plate and streaking onto a new plate as a complex streak three successive times to generate a pure isolate. The isolates were plated for a fourth time on fresh milk plate count agar (MPCA) and incubated aerobically at 30 °C for 3 days. A single colony was picked from these plates and grown in overnight cultures of Luria–Bertani (LB) broth incubated at 37 °C. Stock solutions of these isolates were made by aliquoting 0.5 ml overnight culture to 0.5 ml sterile glycerol (50 %) in a 2 ml sterile screw-top tube and mixed gently before storage at −20 °C.

### 16S ribosomal DNA extraction and sequencing

#### DNA extraction and PCR

According to the manufacturer’s instructions, DNA was extracted from the isolated strains using the GenElute bacterial genomic DNA kit (Sigma-Aldrich). The integrity of the extracted genomic DNA was visualized in a 1 % agarose gel, and the DNA concentration was measured by Qubit (Thermo Fisher Scientific).

The 16S ribosomal DNA was amplified by PCR using Q5 High-Fidelity DNA polymerase 2× master mix (NEB). The amplification program cycle was as described by Frank *et al*. [42] and Lau *et al*. [43], with AGTTTGATCCTGGCTCAG and TACCTTGTTACGACTT primers to amplify almost the entire 16S ribosomal DNA gene. The PCR reactions were made up of 25 µl as per the manufacturer’s instructions and the remainder of the PCR product was cleaned with a Monarch genomic DNA purification kit (NEB). The purified products were Sanger sequenced from both ends using the primers described above (Eurofins).

The Sanger sequences were trimmed for quality, and the forward and reverse reads were aligned using the MegaAlign Pro 17 software from DNASTAR using Clustal Omega as the alignment method to obtain a consensus sequence. The consensus sequence for each isolate was checked against the nucleotide collection (nr/nt) database at the National Center for Biotechnology Information (NCBI), using the blast (Basic Local Alignment Search Tool) network service (http://www.ncbi.nlm.nih.gov).

#### Whole-genome sequencing

Genomic DNA extractions were performed for the isolates using a Monarch gDNA purification kit (NEB) as per the manufacturer’s instructions, and eluted in PCR-grade water. The integrity of the genomic DNA was visualized using 1 % agarose gel, and the DNA concentration was measured by Qubit (Thermo Fisher Scientific). The genomic DNA was sequenced using 2×150 bp paired-end Illumina reads by MicrobesNG. Reads were adapter trimmed using Trimmomatic 0.30 with a sliding window quality cut-off of Q15 [44]. *De novo* assembly was performed using SPAdes version 3.11.1 [45], and the resulting contigs were annotated in Prokka 1.11 [46].

#### Bioinformatic analysis of isolate genomes

To identify the genes responsible for chlorate reduction, the isolates genomes were searched for the presence of chlorite dismutase (*cld*), perchlorate reductase (*pcrA*) and nitrogen reduction pathway-related genes. This was performed individually for each isolate, using the same online blast method mentioned above, except with the *cld*, *pcrA* and nitrogen reduction genes entered in the query sequence section. The *cld* genes checked were those of *Ideonella dechloratans* (AJ296077.1), *Dechloromonas agitata* (AY124796.1), '*Dechlorospirillum'* sp*.* WD (AY540961.1), *Dechloromonas hortensis* (EU436749.1) and *Kocuria rosea* (CP035103.1). The *pcrA* genes checked included *Dechloromonas agitata* (AY180108.1), *Dechlorosoma* sp*.* KJ (AY180108.1), '*Dechlorospirillum'* sp*.* WD (EU273892.1) and *Azospira oryzae* sp*. OGA* (HQ697933.1). The gene sequences encoding the proteins that catalyse nitrogen reduction, specific to the *H. paralvei* sp., were found on the KEGG webpage (https://www.genome.jp/pathway/hpar00910) and included genes encoding: nitrate reductase subunits *narZHI* (AL518_10515, AL518_10520, AL518_10530), nitrate reductase *napAB* (AL518_10920, AL518_10935) and nitrite reductase *nirDB* (AL518_06025, AL518_06030). Following this, strain level comparison was performed using Prokka [46] to annotate and Roary [47] to calculate the pangenome distances. A phylogenetic heatmap was then constructed using the Phandago website version 1.3.0 (https:// github.com/jameshadfield/phandango) [48].

### Analysis

#### Microbial analysis

The spread plate method was used to estimate the total bacterial count of the milk and mineral solution samples. Serial dilutions were prepared in 9 ml maximum recovery diluent, with 100 µl each dilution pipetted onto sterile milk plate count agar (15 ml) and spread using sterile inoculation spreaders (Sarstedt). Plates were incubated for 72 h at 30 °C.

#### Chlorate determination in milk

The quantification of ClO_3_^−^ was performed by HPLC coupled to tandem mass spectrometry (LC/MS-MS) with ESI (electrospray ionization) in negative mode (−ESI). Samples were analysed at the Teagasc laboratories in Dublin, Ireland [16, 49]. The limit of quantification for chlorate was 24 nM in milk.

#### Chlorate determination in selective medium

Chlorate was analysed using a reagent-free ion chromatography (RFIC) system (Dionex) composed of a Dionex ICS-5000 +dual pump and a Dionex ICS-5000 +amperometric/conductometric detector equipped with a temperature-compensated conductivity cell. The eluent was prepared in line with the use of a Dionex Eluent Generator Cartridge (EGC) 500 KOH paired with a Dionex Continuously Regenerated Cation Trap Column (CR-CTC) 500 RFIC (Dionex) for cations and ions, respectively. The anions were separated on an anion-exchange microbore column (IonPac AS19 2×250 mm) fitted with an AG19 (2×50 mm) guard column. Separations were carried out at 30 °C, at a flow rate of 0.25 ml min^−1^, with an injection volume of 25 µl. Chromelian software (version 7.2 SR5; Thermo Fisher Scientific) was used for instrumentation control, data acquisition and processing.

### Statistical analysis

Statistical analysis was performed using spss v26.0 (IBM Statistics). Data sets were analysed for normality using the Shapiro–Wilk test. Analysis was carried out using one-way ANOVA with a post-hoc Tukey test, and non-normal data was analysed using non-parametric tests. *P* values <0.05 were considered significant in all analyses.

## Results and Discussion

### Initial screening for chlorate reduction in milk

**Fig. 2.**
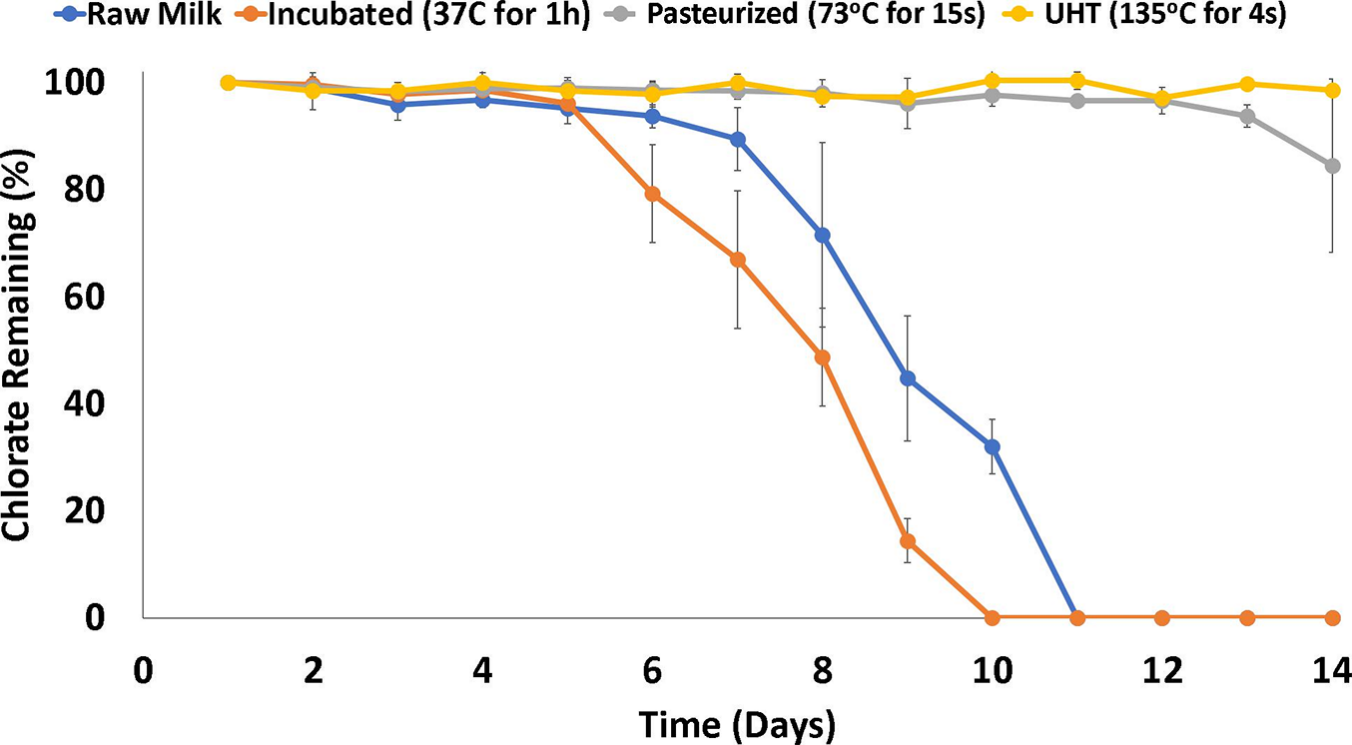
Chlorate degradation (%) relative to milk samples' storage time (days).

Heat treatment’s impact on chlorate degradation relative to the bacterial count in c.f.u. ml^−1^ was monitored over 14 days for samples collected from three discrete farms (Fig. 2). The UHT-treated sample, as sterile control (0 c.f.u. ml^−1^), showed no reduction in chlorate levels, which remained at the original concentration during 14 days of storage. The samples that had been heat-treated using standard pasteurization conditions (73 °C for 15 s) followed a similar trend to the UHT samples, with a decrease in chlorate levels only observed from day 13. There was variation in the per cent chlorate remaining in the milk from the individual farms of 100.77, 62.31 and 90.23 % for farms 1, 2 and 3, respectively, even though milk from farm 2 had significantly lower c.f.u. ml^−1^ value than farms 1 and 3 (*P*<0.05). This differentiation may be due to the microflora being unique to each farm and further confirms that chlorate reduction is not proportional to total bacterial count. These differences could not be explained based on plate counts using skimmed milk agar, as carried out in this experiment, with further detailed species-level investigation requiring a metagenomics approach.

Chlorate levels in unpasteurized (raw) milk began to decrease on day 7, reaching undetectable levels on day 11. In comparison, milk incubated at 37 °C for 1 h to promote microbiological growth prior to storage at 4 °C followed a similar trend to that observed in raw milk but at an accelerated rate, reaching undetectable levels on day 10, compared to day 11 in the raw milk (Fig. 2).

No difference in bacterial growth levels was observed in the chlorate-enriched or control milk samples. This indicates that most of the commensal micro-organisms within the raw milk were not adversely affected by chlorate by either promoting or inhibiting growth to any substantial level, as observed in other studies [50]. However, total bacterial count does not provide species-level differentiation of the microflora present in the milk or the changes therein during the storage period.

The initial screening results identified a microbial degradation of chlorate in milk over time, which has not been reported in the literature to date. The lack of chlorate reduction in the UHT control indicates that chlorate undergoes a minimal chemical reduction in milk and suggests that chlorate reduction is due to microbiological activity, reaching undetectable levels after 10–11 days of storage. This reduction in chlorate levels could be due to DPRBs or respiratory nitrate reductase converting chlorate intracellularly to cytotoxic chlorite [51]. Within a typical DPRB, chlorite dismutase will further break down chlorite to innocuous chlorine and oxygen, minimizing the intracellular build-up of this toxin [26]. However, when respiratory nitrate reductase micro-organisms reduce chlorate, chlorite dismutase is absent, leading to a build-up of cytotoxic chlorite within the cells [51]. Chlorite was not analysed as part of this trial, so it is difficult to ascertain which degradation mechanisms are observed in this study.

There was no significant reduction in chlorate levels during the first week of storage. This was followed by a substantial decrease, with chlorate reaching undetectable levels 4 days after the first observed decrease in chlorate concentration. During this period, the pH decreased due to microbiological growth and metabolism of lactose, with initial chlorate reduction beginning at a pH of ~5.5 and reaching undetectable levels at pH 4.2, a range where chlorate should remain stable [52, 53]. Given the observations in this study, chlorate reduction in milk may depend on the formation of other compounds needed in this reduction pathway, produced through the metabolism of other microbiological processes, acting as a rate-limiting step. It is widely accepted that milk acidification is due to the conversion of the sugar lactose to lactic acid [54, 55], which is the conjugate acid of lactate, a well-studied electron donor for many DPRB organisms during chlorate degradation [56–59].

### Isolation of chlorate respiring bacteria from milk

To isolate bacteria responsible for the chlorate metabolism observed in the initial investigation, milk was inoculated into hungate tubes of SM, chlorate, and either acetate or lactate, with an 0.1 ml aliquot pipetted into fresh medium every 7 days. There was notable turbidity in the initial inoculation for SM acetate and SM lactate tubes, possibly due to casein micelles introduced to the minimal medium in the aliquot of milk, but this observation was reduced with subsequent media changes. The third inoculum showed no visible turbidity in the acetate tubes, with slight cloudiness in the lactate tube, indicating growth.

There was a notable difference between the two media compositions after plating onto the SM chlorate agar containing the respective electron donor (acetate or lactate). There were no colonies on any of the acetate plates but numerous small, creamy-coloured, circular colonies on the lactate-containing plates, which aligns with observations from the literature [26, 58]. Colonies were picked off the plates and purified by the streak plate technique to ensure pure colonies for further characterization.

Growth of these bacteria, specifically on plates containing lactate and not acetate, implies that the former is essential for microbial metabolism of chlorate in this case. This may explain the initial lag in the breakdown of chlorate in the initial trial linked to the acidification of the milk by lactic acid. This was not expected in the trial as acetate is the most common electron donor in documented chlorate degradation, with most DPRB strains able to utilize it [26], with the lack of growth on acetate plates indicating that the bacteria responsible only utilize lactate as an electron donor during chlorate reduction in milk. However, this observation may be linked to the fact that optimal conditions for acetate metabolism may not have been defined.

The authors are unaware of any other attempts to isolate a (per)chlorate-reducing bacteria from milk but are aware of other studies noting perchlorate reduction by microbes in the rumen. The isolation of DPRB from these studies has been unsuccessful [38, 39, 60]. However, DPRBs have been isolated from various environments, including swine waste, aquatic sediment, soil, paper mill sediment and petroleum-contaminated soil [29, 61].

#### Analysis of DNA extracted from isolates

After isolation and purification on SM and skimmed milk agar plates, five isolates were chosen for sequencing based on colony morphology and enrichment conditions. Isolates A and D originated from the milk spiked with chlorate for 7 days before inoculation into SM lactate tubes, and isolates B, C and E were from fresh raw milk inoculated into SM lactate tubes. Isolates were found to have genome sizes from 4.75 to 4.92 Mbp, and DNA G+C contents ranging from 48.03 to 48.13 mol % were observed for the sequenced strains, with assembly statistics described in Table 1

**Table 1.** Assembly statistics of five *H. paralvei* isolates sent for whole-genome sequencing

Isolate	No. of reads with insert size >300 bp	No. of contigs ≥0 bp	No. of contigs ≥1000 bp	Total length ≥0 bp (bp)	Total length ≥1000 bp (bp)	No. of contigs	Largest contig (bp)	Total length (bp)	G+C (mol%)
A	952 303	121	20	4 822 587	4 781 663	46	1 771 544	4 798 243	48.01
B	545 496	66	33	4 934 699	4 924 452	40	815 785	4 929 610	48.13
C	1 245 373	57	22	4 800 798	4 789 901	28	1 146 023	4 794 319	48.09
D	1 992 554	103	30	4 778 480	4 755 446	39	1 215 454	4 761 959	48.03
E	2 307 909	100	31	4 777 635	4 754 488	43	1 215 454	4 762 718	48.03

**Fig. 3.**
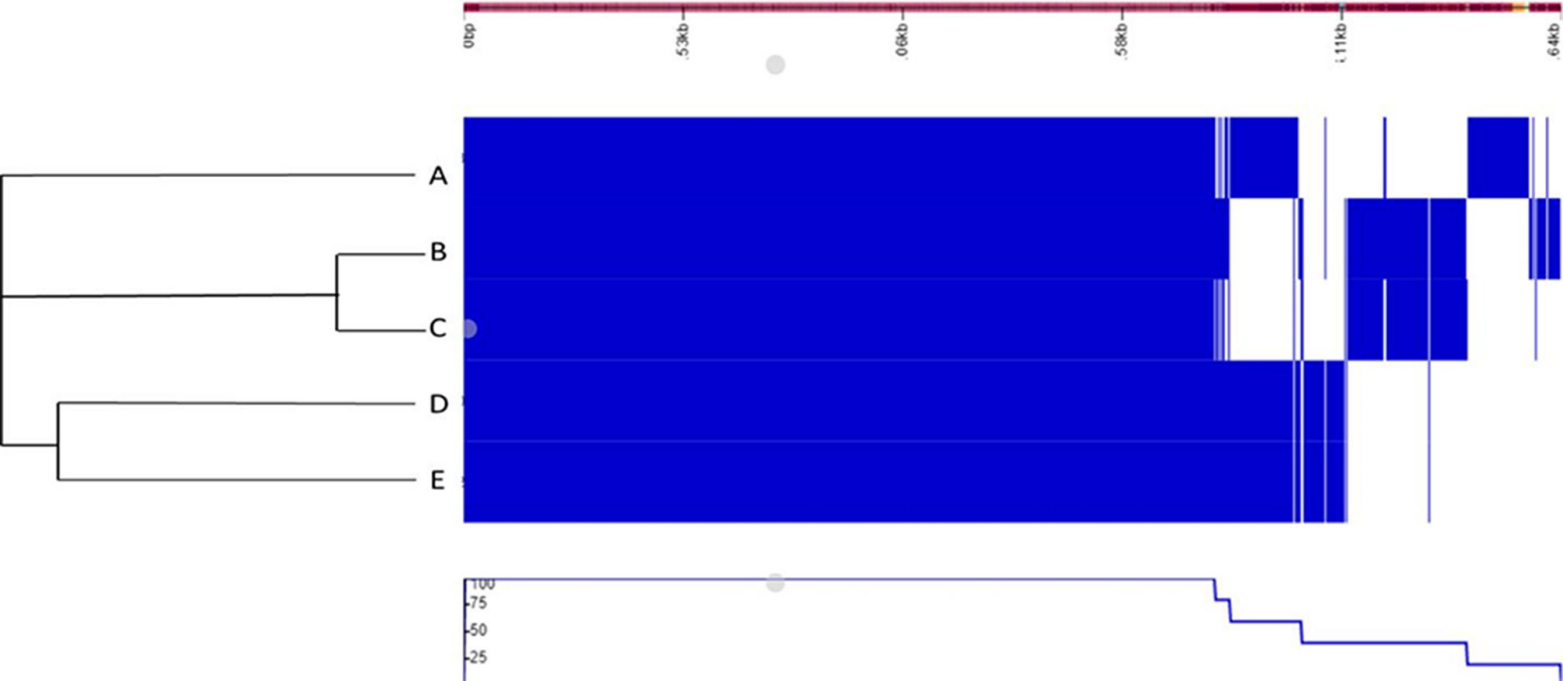
Dendrogram showing clustering of digitally inverted fingerprints of five isolated strains using media for the selective growth of chlorate-reducing bacteria.

Comparative genome analysis indicated that isolates B and C and D and E were most closely related (Fig. 3). Isolates B, C and E were isolated from raw milk inoculated into SM on the day of collection, and isolates D and A were from milk spiked with chlorate and refrigerated for 7 days before inoculation into SM. The high similarity between isolates D and E indicates that they could be the same bacterial strain, isolated from two different enrichment approaches. All five isolates sent for genomic analysis were determined to be *Hafnia paralvei* species of the family *Hafniaceae *. Genomic data and annotation of genes did not highlight any genes typically associated with the DPRB pathway.

#### Bioinformatic analysis of isolate genomes

Analysis of the genes within these isolates highlighted that they are all dissimilatory nitrate-reducing (DNR) bacteria, with three genes encoding proteins that catalyse nitrate reduction to ammonia. First, through the reduction of nitrate to nitrite by either membrane-associated nitrate reductase (*narZHI*) or periplasmic nitrate reductase (*napAB*). Following this, nitrite is reduced to ammonia by nitrite reductase (*nirDB*). Numerous studies on the genera *Escherichia *, *Aerobacter *, *Pseudomonas* and *Hafnia* show that nitrate reductases found in DNR bacteria can reduce chlorate [51, 62]. Of the two nitrate reductases, Nar is more responsible for chlorate reduction than Nap, which plays a secondary role in chlorate reduction by first helping the bacterial cell establish an anaerobic environment and then reducing chlorate to a small extent [63, 64]. Knockout studies also show that mutants lacking the *nar* nitrate reductase gene lose their ability to reduce chlorate [65–67]. Based on our findings and existing literature, the nitrate reductase Nar, specifically NarZHI in the genus *Hafnia *, can be expected to be the prime candidate responsible for chlorate reduction in the *H. paralvei* isolates obtained. However, there may be other factors involved. Variations in methionine oxidation patterns in the proteomes of chlorate-stressed bacterial cells have been suggested by literature as another possible mechanism influencing chlorate metabolism, but this link is yet to be fully characterized and understood [66, 68]. The activity of nitrate reductases, and so its chlorate-reducing ability, has also been identified to be influenced by various factors, including the enzymes involved downstream in the DNR pathway, such as the nitrite reductase Nir, and the proteins involved in quorum sensing such as LasR, and gene regulation of the nitrogen mobility and metabolism pathways [62, 67]. The reduction of chlorate by nitrate reductase, both Nar and Nap, produces chlorite, which is cytotoxic to micro-organisms, which is important to note as DNR bacteria lack chlorite dismutase [69]. Still, the production of chlorite was not a focus of this study, so it is impossible to deduce whether there is an alternative means of mitigating chlorite build-up or whether nitrate reductase is responsible for the metabolism of chlorate in milk.

#### Growth and chlorate metabolism of isolates

**Fig. 4.**
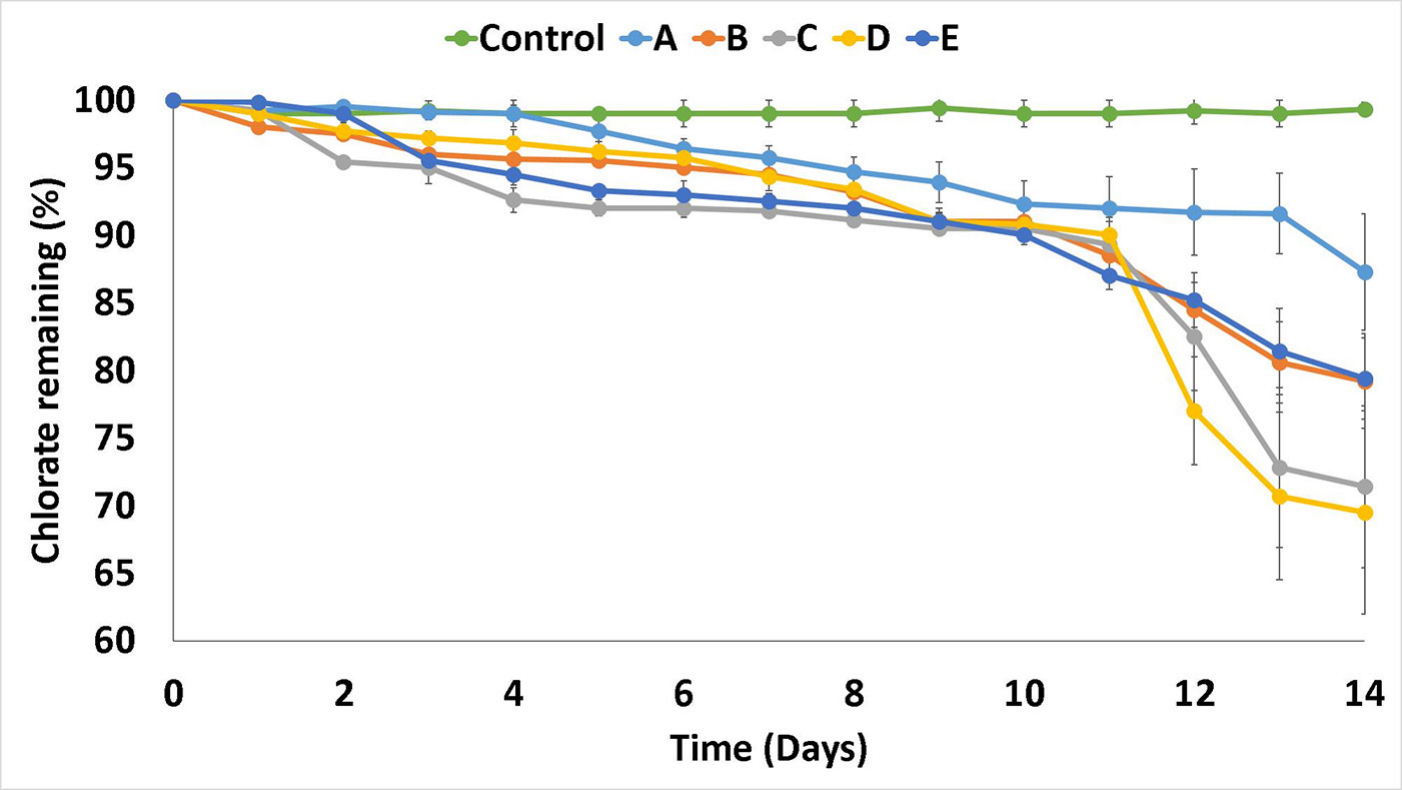
Chlorate concentration remaining (%) versus time (days) for isolates grown in selective medium containing chlorate and lactate under anaerobic conditions.

Purified isolates were anaerobically inoculated into fresh SM containing 500 mg chlorate l^−1^ (6 mM) for 14 days, and the bacterial count and chlorate degradation were monitored daily. There was no detectable decrease in chlorate in the sterile control, indicating that chlorate within the medium remained stable. All isolates demonstrated an ability to significantly break down chlorate within the medium over the 14 day incubation period (*P*<0.05) (Fig. 4). There was a significant difference in chlorate reduction at day 10 (*P*<0.05), and an increase in chlorate reduction was measured for isolates C and D in particular. The increase in chlorate degradation is likely linked to the logarithmic growth of the isolates within the medium, resulting in a much greater number of bacteria and, thus, a higher level of enzyme activity in the solution.

Compared to the degradation of chlorate in milk, there was a significant difference in the breakdown of chlorate relative to the starting concentration (*P*<0.05), with none of the isolates reducing chlorate to undetectable levels. The lower level of chlorate removal could be due to the cultures in this study being incubated with a much higher chlorate concentration of 6 mM, which was chosen as a standard concentration based on previous studies [32, 41], compared to 1.2 mM used during the initial experiments. This is supported by the study of Simon and Weber [50], who found that levels of perchlorate greater than 2 mM inhibited chlorate reduction while lower concentrations did not.

**Fig. 5.**
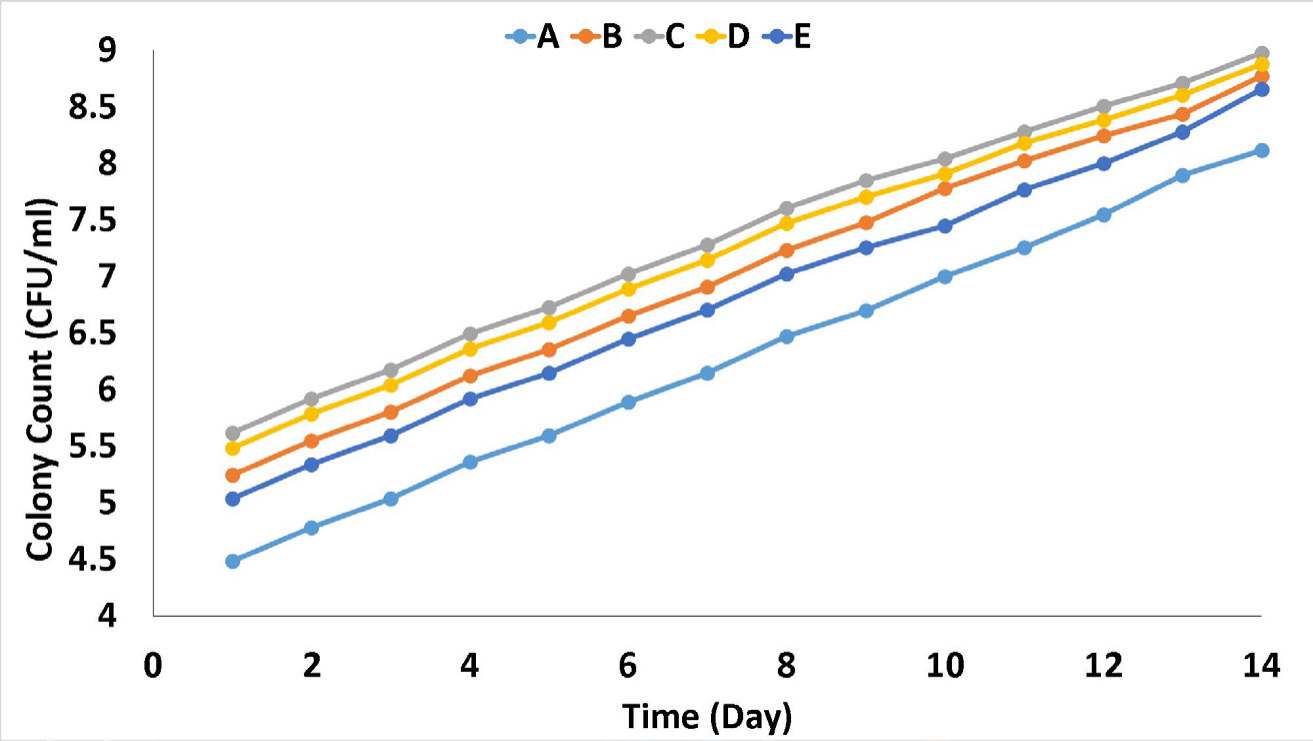
Colony count (log c.f.u. ml^−1^) versus time (days) for isolates grown in selective media containing chlorate and lactate under anaerobic conditions.

All isolates grew in the medium over the 14 days (Fig. 5) (single tube taken per day per isolate with a new, unopened tube taken daily). This indicates that chlorite did not increase to toxic levels within the inoculated tubes, indicating the presence of chlorite degradation mechanisms, as discussed in earlier sections.

This study presents the future potential of the *H. paralvei* isolates, or their purified chlorate-reducing enzymes, to naturally reduce chlorate residues in milk or chlorate-contaminated dairy processing wastewater. However, further investigations on the precise mechanisms by which these DNR bacteria are reducing chlorate need to be performed, involving knockout, up- or down-regulation of the nitrate reductase *narZHI* and *napAB* genes. Applying these isolates or the nitrate reductase enzymes may aid in decreasing the environmental impact of chlorate discharge in dairy wastewater, similar to water treatment, where DPRBs are used for perchlorate remediation [70]. It was demonstrated that milk microflora could completely break down residual chlorate contamination of 1.2 mM to undetectable levels in 2 weeks, with higher concentrations of 6 mM having an apparent inhibitory impact on the ability to break this contaminant down completely. The expected levels of chlorate in wastewater would be much lower than the 6 mM used during the isolation and characterization steps of this study, due to the regulatory limits established for water being 3 mM within the European Union, so it is possible that these bacteria completely degrade chlorate in dairy wastewater streams without inhibition.

### Conclusion

Chlorate was observed to be degraded naturally over time in unpasteurized milk, reaching undetectable levels within 11 days. In comparison, UHT and pasteurized milk samples demonstrated no significant levels of degradation during storage. Isolation techniques utilizing lactate- and chlorate-enriched medium identified five *H. paralvei* isolates, a species more commonly found in milk from farms where chlorine sanitizing agents are used, further highlighting the relationship between *Hafniaceae* and chlorine and its associated disinfection by-products (chlorate). As lactate was identified as an electron donor that promoted chlorate reduction, it is hypothesized that the initial microbial activity in raw milk, which would be expected to generate lactic acid, is a rate-limiting mechanism in this reaction, as chlorate breakdown before day 7 was not significant. Genomic data and annotation of genes did not highlight any genes typically associated with existing DPRBs. Therefore, the nitrogen reduction pathway within these *H. paralvei* may be responsible for chlorate metabolism. However, further studies are required to determine the exact mechanisms involved in this chlorate reduction. These bacteria, or their purified enzymes, may have industrial applications in the future for the remediation of chlorate in milk or for the treatment of dairy effluent streams, mitigating environmental contamination by chlorate.
